# The Influence of Disabilities in Activities of Daily Living on Successful Aging: The Role of Well-Being and Residence Location

**DOI:** 10.3389/fpubh.2019.00417

**Published:** 2020-01-28

**Authors:** Xiaoshan Li, Jingjing Wang, Shenghong Dong, Jianping Fu, Jianping Liu

**Affiliations:** ^1^School of Psychology, Jiangxi Normal University, Nanchang, China; ^2^Center of Mental Health Education and Research, School of Psychology, Jiangxi Normal University, Nanchang, China; ^3^College of Life Science, Jiangxi Normal University, Nanchang, China

**Keywords:** mortality, cognition impairment, disability in activities of daily living (ADLs), well-being, residence place

## Abstract

This study aimed to investigate the effect of disability in activities of daily living (ADLs) on successful aging, and the possible moderators between them. Based on data from the Chinese Longitudinal Healthy Longevity Surveys (CLHLS), we used the Cox proportional hazards model of survival analysis and multivariate regression analysis (SPSS 16.0) to test our hypotheses. Mortality or cognition impairment were the dependents variables, and disability in ADLs was the independent variable. Well-being and residence location were the moderators. The results showed that in survey 2005, the Chinese elders with disability in ADLs, after controlling elder's gender, age, residence place, and marital status, often experienced more mortality and cognition impairment risk in the next 3 years. Our findings also showed that the increased mortality risk among elders with disability in ADLs was lower in those with higher well-being or younger age. The increased cognitive impairment risk among elders with disability in ADLs was lower in those living in the city than those living in a rural area or small towns. These findings contribute to a better understanding of the relationship between disability in ADLs and successful aging among Chinese elders. Our findings also expose other elements to consider such as psychological factors (e.g., well-being) and residence location in the relationships between the disability in ADLs and mortality (or cognition impairment), which have a psychological impact in successful aging of the Chinese elders.

## Introduction

An aging population is a significant challenge for most countries in the world, including China (1). Attention should be paid on aging population due to their physical decline, high mortality, possible depression symptom, and cognition impairment, which are regarded as important obstacles in reaching the goals of successful aging (2–5). The activity center was proved as an effective model for health support and health-related quality of life improvements for older people (6, 7). However, the direct impact of disability in activities of daily living (ADLs) on longevity and cognitive impairment and how the relationship varies with other variables are still unclear.

Disability in ADLs is defined as individuals who are partly or entirely unable to perform basic ADLs (e.g., dressing) (1, 8). Elders with disability in ADLs often need help from their family members or caregivers (9). Due to the declining ability in making their own choice and shrinking social network (2, 5), elders often experience low self-control and a strong sense of loneliness or meaninglessness, which are risk factors that increase elder's cognition impairment, suicide, and mortality risk (10–14). So disability in ADLs might have a direct effect on the elder's mortality and cognitive function. Besides, the association between disability in ADLs and mortality (or cognitive impairment) can vary due to individual mental status (e.g., well-being) and living environment (e.g., residence place). Comparing with low well-being elders or those living in Chinese rural area, elders with high well-being or those living in Chinese urban area might have high internal (e.g., positive attitude to life) or external resources (e.g., social or medical support) against adversity (e.g., disability in ADLs) (1, 10, 12, 15), which could reduce the increased risk of disability in ADLs on elder's mortality or cognitive impairment. Thus, residence place and well-being could moderate the relationship between the disability in ADLs and cognitive impairment (or mortality). Therefore, two hypotheses were proposed as follows.

*Hypothesis 1:* The disability in ADLs was positively related to mortality and cognitive impairment.

*Hypothesis 2:* The relation between the disability in ADLs and mortality or cognitive impairment might be moderated by well-being or residence place.

## Materials and Methods

### Participants

This study used data from the 2005 and 2008 Chinese Longitudinal Healthy Longevity Surveys (CLHLS). CLHLS is a national survey which adopted a targeted random-sample design to ensure the representativeness of the Chinese aging population considering the gender, age, and other factors. In survey 2005, 15,638 elders participated, of those participants, 2,938 were lost in the following 3 years (2008 survey) and were excluded. Therefore, we only used data from 12,700 participants in the analysis. The age of participants (42.7% men, *n* = 5,430) ranged from 60 to 112 years (M = 86.1, SD 11.7), 3,871 participants (30.5%) were married and living with their spouses, 2,461 participants (19.4%) received pension as retired workers or government officers (economic status), 2,168 participants (21%) experienced serious physical or mental illness (e.g., cancer, dementia) in the previous years before survey 2008, and 2,795 participants' (23%) had their residence in cities. The Research Ethics Committees of Peking University and Jiangxi Normal University reviewed the study protocol and granted approval for the Protection of Human Subjects for CLHLS, including the collection of data used in the study.

### Measurements

Cognition impairment was measured with the mini-MMSE (16), which is proved as an excellent assessment tool for Chinese elders (17). The mini-MMSE includes 24 items regarding attention, calculation, recall, and language, with a total score ranging from 0 to 30, with higher scores indicating less cognitive impairment. For mortality, a score of 0 indicates that the participants were still alive in survey 2008, while a score of 1 indicates that the participant was dead. Well-being was measured using a psychological well-being scale (12), which includes seven items covering positive effects (e.g., optimism) and adverse effects (e.g., loss of self-worth) with a five-point Likert scale. A psychological well-being index was conducted using the total scores of these seven items, with higher scores indicating better well-being. Disability in ADLs was measured using a brief scale of disability in ADLs, which includes six items (e.g., eating) and proved to be a proper measurement for Chinese elder's functional capacity (1). A score of 1 indicates that the participants need assistance with the daily activities, while a score of 0 indicates that participants are self-sufficient and do not need any assistance.

Gender, martial status, economic status (e.g., received pension as retired workers or government officers), and serious illness were proved to have an impact on elder's mortality and cognition impairment (1, 13, 18) and were used as control variables.

### Procedure Process and Analytical Strategies

All the variables in the present study were collected in survey 2005, except the data of cognitive impairment and mortality, which was collected in survey 2008. To examine the relationship between disability in ADLs and mortality, we used two Cox proportional hazards model of survival analysis (19). Model 1 included the independent variable (e.g., disability in ADLs), moderators (e.g., residence place, well-being), and the control variables (e.g., economic status, serious illness, marital status, and gender). Model 2 added a two-way interaction between disability in ADLs and residence place, disability in ADLs and well-being. In the survival analysis, survival time for survivors (or decreased respondents) was the number of days between the date of survey 2005 and the date of survey 2008 (or death). Two multiple variables regression models were used to measure the relationship between disability in ADLs and cognitive impairment. Model 3 included the independent variable, moderator, and control variables, as showed in model 1. Model 4 added a two-way interaction between the independent variables and moderators, as showed in model 3. In model 2 and 4, we did not include the two-way or three-way interaction between disability in ADLs and other control variables (except age in model 2 of Table 1) because they were not significant. All models above were analyzed using the SPSS software (version 16.0).

**Table 1:** The relative risk factors of mortality among Chinese elders (*N* = 12,700).

**Variables**	**Mortality**			
	**Model 1**		**Model 2**	
	**HR (CI)**	***P*-value**	**HR (CI)**	***P*-value**
**MAIN TERMS**				
Disability in ADLs (No)	1.35 (1.32, 1.39)	<0.001	1.04 (0.89, 1.20)	0.64
**TWO-WAY INTERACTIONS**				
Disability in ADLs^*^Residence place			1.01 (0.96, 1.07)	0.70
Disability in ADLs^*^well-being			1.02 (1.01, 1.03)	<0.001
Disability in ADLs^*^age			0.08 (0.07, 0.08)	<0.001
**CONTROLS**				
Didn't receive pension (receive)	1.34 (1.27, 1.42)	<0.001	1.34 (1.26, 1.42)	<0.001
Current married (No)	0.36 (0.34, 0.37)	<0.001	0.36 (0.34, 0.37)	<0.001
Serious illness (No)	1.08 (1.02, 1.13)	<0.01	1.07 (1.02, 1.13)	<0.01
Age in survey 2005	1.31 (1.24, 1.39)	<0.001	1.30 (1.24, 1.38)	<0.001
Men (Women)	1.02 (0.99, 1.04)	0.10	1.02 (0.99, 1.04)	0.09
Low well-being (high)	1.02 (1.01, 1.03)	<0.001	0.99 (0.97, 1.01)	0.26
Rural (city)	1.07 (1.04, 1.10)	<0.001	1.06 (1.01, 1.11)	<0.05

*HR, relative risk; CI, confidence interval*.

## Results

### Mortality

Table 1 shows the relative risk factors of mortality. Model 1 shows that one-point increase in disability in the ADLs index was associated with an increased hazard ratio (HR) of 35% (HR = 1.35, 95% CI: 1.32–1.39), after controlling for elder's gender, age, residence place, and well-being. Model 2 shows that the HR of the interaction between disability in ADLs and well-being was more than 1 (HR = 1.02, 95% CI: 1.01–1.03), while the HR of the interaction between disability in ADLs and age was < 1 (HR = 0.08, 95% CI: 0.07–0.08). This indicates that the increased mortality risk among elders with disability in ADLs was lower in those with higher well-being (or younger age) than in those with lower well-being (or older age).

### Cognition Impairment

Table 2 shows the relative risk factors of cognitive impairment. Model 3 shows that elders with disability in ADLs had a worse cognitive function (β = −0.06, *P* < 0.001), after controlling for elder's gender, age, residence place, and well-being. There is a significant interaction effect between disability in ADLs and residence place in predicting elder's cognitive function (β = −0.24, *P* < 0.001). Simple slope analysis shows the increased cognitive impairment risk among elders with disability in ADLs was lower for those living in the city than in those living in rural areas or small towns (city: β = −0.04, *P* = 0.24; rural or small towns: β = −0.084, *P* < 0.001).

**Table 2:** The relative risk factors of cognitive impairment among Chinese elders (*N* = 12,700).

**Variables**	**Cognitive function**			
	**Model 3**		**Model 4**	
	**β**	***P*-value**	**β**	***P*-value**
**CONTROL**				
Economic status	0.08	<0.001	0.1	<0.001
Marital status	−0.02	0.40	−0.01	0.45
Serious illness	−0.02	0.17	−0.02	0.18
Age in survey 2005	−0.30	<0.001	−0.32	<0.001
Gender	−0.14	<0.001	−0.13	<0.001
Well-being	0.02	0.22	−0.02	0.61
Residence place	−0.05	<0.01	−0.25	<0.001
**MAIN TERMS**				
Disability in ADLs	−0.06	<0.001	−0.14	0.09
**TWO-WAY INTERACTIONS**				
Disability in ADLs^*^Residence place			−0.24	0.001
Disability in ADLs^*^well-being			0.09	0.26
Disability in ADLs^*^age			0.02	64
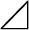 *R*^2^	0.15		0.02	

*For cognitive function: high score means better cognitive function; disability in ADLs: 0 = No, 1 = Yes; economic status: 0 = didn't receive pension, 1 = receive; marital status: 0 = current married, 1 = widows; serious illness: 0 = No, 1 = Yes; Gender: 0 = men, 1 = women; well-being: high score means higher well-being; residence place: 0 = city, 1 = rural area or town*.

## Discussion

The study findings show that in 2005, Chinese elders with disability in ADLs often experienced more mortality and cognition impairment in the following 3 years, after controlling for age, residence place, etc. (supported hypothesis 1). It indicates that disability in ADLs is a risk factor for elder's successful aging due to the shrinking social network and the declining ability in making their own choice. Our finding also found that the increased mortality risk among elders with disability in ADLs was lower in those with higher well-being (or younger age). A possible reason might be that high well-being (or younger age) act as protective factors and could provide enough mental (or physical) energy to reduce the risk of disability in ADLs. Moreover, we found that the increased cognitive impairment risk among elders with disability in ADLs was lower in those living in cities than in those living in rural areas or small towns. The possible explanation is that living in cities could provide better medical or educational resource, which could reduce the risk of disability in ADLs on cognitive impairment while living in rural areas or small towns could not. These findings suggest that government officers should pay more attention to Chinese elders with disability in ADLs, especially for those who lived in town or village in policy-making for successful aging. And these findings also suggest that psychological factor (e.g., well-being) can be used as a psychological intervention for successful aging among Chinese elders.

Although survival time could reflect the longevity, the valid age of death might be a better index for measuring longevity. Future research could use the valid age of death as the index of longevity to provide a robust evidence for the relation between disability in ADLs and successful aging. In addition, Brain derived neurotrophic factors (BDNF) and pro-inflammatory cytokines (e.g., Interleukin-1 beta) are implicated in cognitive impairment and dementia (7, 20). And medical conditions or comorbidities, and education also play important role in the process of the successful aging (5, 19). Further research should focus on other factors which were not explored in this study, such as the direct effect of BDNF, medical conditions or comorbidities, and interaction effect of BDNF, medical conditions or comorbidities, education, and disability in ADLs.

## Conclusion

Our findings showed that disability in ADLs is a risk factor of mortality and cognition impairment among Chinese elders, and the increased mortality or cognitive impairment risk of disability in ADLs could be moderated by the variables of well-being, age, or residence place.

## Data Availability Statement

Publicly available datasets were analyzed in this study. This data can be found here: http://opendata.pku.edu.cn/dataverse/CHADS.

## Ethics Statement

This study was carried out in accordance with the recommendations of the Research Ethics Committees of Peking University with written informed consent from all subjects. All subjects gave written informed consent in accordance with the Declaration of Helsinki. The protocol was approved by the Research Ethics Committees of Peking University.

## Author Contributions

XL, JF, and JL participated in the design of this study, carried out the data acquisition, analysis, manuscript editing, critical revision of the manuscript for important intellectual content, and final approval of the version to be published. JW and SD carried out the literature search, contributed to data acquisition, and manuscript editing. XL provided the largest contribution to the manuscript. All authors approved the final version of this manuscript and agree to be accountable for all aspects of the work.

### Conflict of Interest

The authors declare that the research was conducted in the absence of any commercial or financial relationships that could be construed as a potential conflict of interest.
